# Biotechnology Approaches in Food Preservation and Food Safety

**DOI:** 10.3390/foods11101391

**Published:** 2022-05-11

**Authors:** Joana Barbosa, Paula Teixeira

**Affiliations:** Universidade Católica Portuguesa, CBQF—Centro de Biotecnologia e Química Fina—Laboratório Associado, Escola Superior de Biotecnologia, Rua de Diogo Botelho 1327, 4169-005 Porto, Portugal

Driven by different motivations, we have seen a rise in the demand for “natural”, “preservative-free”, and “clean label” foods. Simultaneously, consumers are expecting safer and also more convenient foods with long shelf lives. This is undoubtedly one of the great challenges of the food industry and an opportunity for “Biotechnology Approaches”.

This Special Issue of *Foods*, entitled “Biotechnology approaches in Food Preservation and Food Safety”, was devoted to topics focused on biotechnology approaches in the pre- and post-harvesting of produce, food processing and preservation technologies, and methods for monitoring food safety and quality. Subsequent to the peer review process, three original research articles and three reviews were included in this Special Issue of *Foods*.

Biotechnological approaches are an alternative to the chemical synthesis of many compounds of interest. In Sharma et al. [1], researchers from Punjabi University (India), Hiroshima University (Japan), and King Saud University (Saudi Arabia) describe the development of a laboratory-scale bioprocess for high-level fermentative production of L-alanine using metabolically engineered *Pediococcus acidilactici* BD16 (alaD+). L-alanine is a nonessential amino acid with a wide range of food, pharmaceutical, and veterinary applications. According to the authors, “the novelty of the study is the use of a synthetic system biology-mediated metabolic engineering strategy that abolished the need of complex gene knock-outs for engineering a microbial strain, which may hinder the maximal expression of the desired cloned gene.” The authors suggested that the developed bioprocess using recombinant *P. acidilactici* BD16 (alaD+) could be the best alternative to the chemical-based commercial synthesis of L-alanine for potential industrial applications.

Studies by Keli et al. [2] from the University of South Florida and the University of Florida, USA, demonstrated that reducing fungicide applications combined with proper handling during the supply chain reduces costs, fruit waste, and maintains overall strawberry quality. This study also reinforced the need to wash strawberries to reduce both the microbial load and the residual fungicide levels.

Gomes et al. [3] from CBQF, Portuguese Catholic University (Portugal), highlighted the need for defined standard methods to determine the minimum inhibitory concentrations of natural antimicrobial compounds. The authors reported that some microorganisms were inhibited by the natural compounds tested using the agar dilution method but not inhibited by the same compounds and at the same concentrations using the drop diffusion technique. In the vision of the authors, “the use of standard techniques such as those used for antimicrobials of clinical applications are crucial to compare results obtained in different studies and different matrices”.

Pinto de Rezende et al. [4], from the same institution, presented a literature review on techniques to preserve shellfish as an alternative to refrigeration, focusing on the application of biodegradable films and coating technology; superchilling; irradiation; high-pressure processing; hyperbaric storage; and biopreservation with lactic acid bacteria, bacteriocins, or bacteriophages. According to the authors, “although no technique appears to replace refrigeration, the implementation of additional treatments in the seafood processing operation could reduce the need for freezing, extending the shelf life of fresh unfrozen products”.

The use of lactic acid bacteria (LAB) as biopreservatives has been gaining interest, since they are “generally recognized as safe” and have antibacterial and antifungal activities due to the colonization and competition for nutrients and space or the production of antimicrobial metabolites such as lactic acid, ethanol, and bacteriocins. Martín et al. [5], from the Universidad de Extremadura (Spain), conducted a review on LAB-based strategies to control *L. monocytogenes* in ready-to-eat meats and dairy-ripened products. These researchers stated that “The combination of selected LAB strains with antimicrobial compounds, such as acid/sodium lactate and other strategies, as the active packaging could be the next future innovation for eliminating the risk of *L. monocytogenes* in meat and dairy-ripened products.”

De Oliveira et al. [6], from Federal University of Paraíba, Brazil, presented a cutting-edge study on the development and application of probiotic-loaded edible films/coatings to preserve fresh and minimally processed fruit and vegetables. The primary materials used for the formulations, their effects on the survival of probiotics and the impacts of their application on the quality, safety, and storability of the coated fruit and vegetables were addressed. This is a matter of the utmost relevance as the number of severe outbreaks associated with fresh produce has been increasing and fruit and vegetable wastage between primary production and the final consumer represents between 35% and 55% of the production volume.

In summary, the different papers published in this Special Issue address different “Biotechnology Approaches” in the food sector and highlight the need for more research in this field. We thank the authors for their valuable contributions to this Special Issue, which we hope readers will find interesting and instructive.
